# Barriers to Working With National Health Service England’s Open Data

**DOI:** 10.2196/15603

**Published:** 2020-01-13

**Authors:** Seb Bacon, Ben Goldacre

**Affiliations:** 1 The DataLab Nuffield Department of Primary Care Health Sciences University of Oxford Oxford United Kingdom

**Keywords:** informatics, health services, software, access to information

## Abstract

Open data is information made freely available to third parties in structured formats without restrictive licensing conditions, permitting commercial and noncommercial organizations to innovate. In the context of National Health Service (NHS) data, this is intended to improve patient outcomes and efficiency. EBM DataLab is a research group with a focus on online tools which turn our research findings into actionable monthly outputs. We regularly import and process more than 15 different NHS open datasets to deliver OpenPrescribing.net, one of the most high-impact use cases for NHS England’s open data, with over 15,000 unique users each month. In this paper, we have described the many breaches of best practices around NHS open data that we have encountered. Examples include datasets that repeatedly change location without warning or forwarding; datasets that are needlessly behind a “CAPTCHA” and so cannot be automatically downloaded; longitudinal datasets that change their structure without warning or documentation; near-duplicate datasets with unexplained differences; datasets that are impossible to locate, and thus may or may not exist; poor or absent documentation; and withholding of data for dubious reasons. We propose new open ways of working that will support better analytics for all users of the NHS. These include better curation, better documentation, and systems for better dialogue with technical teams.

## Background

Open data is briefly defined as data that anyone can access, use, modify, and share; more technical definitions are available from various sources [1]. Open data must, therefore, be shared in the public domain or provided under an open license, accessible and downloadable without charge, provided in a form that is machine readable, and provided in an open format, which itself places no restrictions on use.

The UK government has long recognized that simply publishing data is, in itself, not sufficient to meet these criteria and also not sufficient to drive change and innovation. The 2012 Open Data White Paper set out 14 information principles declaring that data should be easy to find, available without registration, and accompanied by meaningful descriptive text, alongside various other more technical recommendations [2]. It also adopted the 5-star scheme from Tim Berners-Lee for assessing the extent to which datasets are truly reusable: this ranges from unstructured proprietary documents through to fully linked data in nonproprietary formats with uniform resource identifiers. Although a swathe of data has been licensed appropriately for free reuse, the more detailed principles outlined in the White Paper have not been consistently adopted.

The National Health Service (NHS) in England has long agreed that transparency can lead to better outcomes for patients and taxpayers [3,4], and the Department of Health first requested publication of some prescriptions data as early as 1998 [5]. However, to our knowledge, there has been very little work describing how open health data are used in practice by NHS analysts, industry, or health researchers; and no prior work on the barriers was encountered.

Our group develops and maintains OpenPrescribing.net, an online and publicly accessible tool, to help users explore highly granular NHS primary care prescribing open data. It is widely used with over 15,000 unique users each month. Its users are predominantly from within the NHS, but industry and patient groups are also well represented. In England, the planning and commissioning of health care services for each local area is carried out by Clinical Commissioning Groups (CCGs), who alongside NHS England commission primary care services from individual general practitioner (GP) practices. GPs have considerable freedom in prescribing behavior, with the costs of prescriptions usually being borne by CCGs. The transfer of money from CCGs to pharmacies (and other organizations) who dispense prescriptions to patients is mediated by the NHS Business Services Authority (NHSBSA), which processes all prescribing transactions to determine correct payments. The NHSBSA is, therefore, also responsible for converting data submitted from pharmacies into a standard format. Although it exists for an economic purpose, the existence of this very high-quality dataset provides a unique opportunity to find ways to improve the quality, safety, and cost-effectiveness at all individual GP practices across England. Our tools support complex bespoke data queries alongside numerous predefined standard measures for safety, cost, and effectiveness. In total, 92.1% (176/191) of CCGs are signed up to monthly alerts, which automatically identify high-priority action items. We have published peer-reviewed research showing that prescribing is substantially improved in practices and CCGs where OpenPrescribing.net data are accessed [6].

OpenPrescribing.net is built on top of data that are theoretically publicly accessible. We have repeatedly encountered time-consuming barriers to accessing and processing these data. In this paper, we have described some of these barriers and made recommendations on how the NHS could share data more effectively.

The views set out below are informed by our technical work building OpenPrescribing.net but also by our broader background. The DataLab at the University of Oxford is a mixed team of software engineers, clinicians, academics, and analysts turning NHS data into tools and services to directly improve patient care. We aim to pool skills and combine best practices from software engineering and academia, producing open source software, open prototypes, and open workbooks. On GitHub, under open licenses, we have shared 44,000 lines of code in 34 public repositories with over 5000 commits; 850 Python files; 105 Structured Query Language (SQL) files containing 4600 lines of SQL; 140 Jupyter notebooks; and over 1000 GitHub issues, each containing detailed descriptions of specific problems we have encountered and their technical solutions. Many of us have also worked previously in organizations that promote open access to knowledge. In more concrete terms, as reference to our experience of working with NHS open data, at least 8 different datasets must be located, downloaded, converted, normalized, interpreted, combined, and then processed to create even 1, apparently simple, mapped insight on OpenPrescribing.net: “over the past 5 years, NHS North Cumbria spent £63,000 on Linaclotide.”

In the following section we have described a range of barriers we have encountered in accessing NHS open data. For each problem domain we describe the datasets we are aiming to access, the barriers encountered, and some suggested solutions that would make the data usable and impactful.

## Problems With the Prescribing Data Itself

Each month, we download and process prescribing data for NHS England. The best practice [7-9] is that this should be easily discoverable, accessible without human intervention, made available at addresses that do not change, and documented so the relevant concepts are clearly explained. None of these are entirely true of the prescribing data.

For example, consistently locating the data is difficult: both initially and on an ongoing basis with each new month of data. The first challenge for a consumer of the data is picking a dataset to use. A total of 2 very similar datasets are published by 2 different organizations: NHS Digital, and NHSBSA. The NHS Digital dataset is published on the first Friday of the third month after data collection, whereas the NHSBSA dataset is usually available 6 weeks following data collection. Neither of these datasets reference the other in their documentation, and we have found no single location that identifies them as complementary sources.

Until 2017, we used NHS Digital’s version (known as practice level prescribing data), simply because this is the easiest to find. For 2 years we retrieved the data from NHS Digital’s data repository [10] but that link broke during 2018; following a content reorganization, it is now available on a new NHS Digital website [11]. Complicating easy discovery of this dataset is the fact that it is also listed in NHS England’s *Data Catalogue* but only with data up to May 2016 [12]. Every time the location of the data changes, it breaks the software we have written to automatically download it.

**Figure 1:**
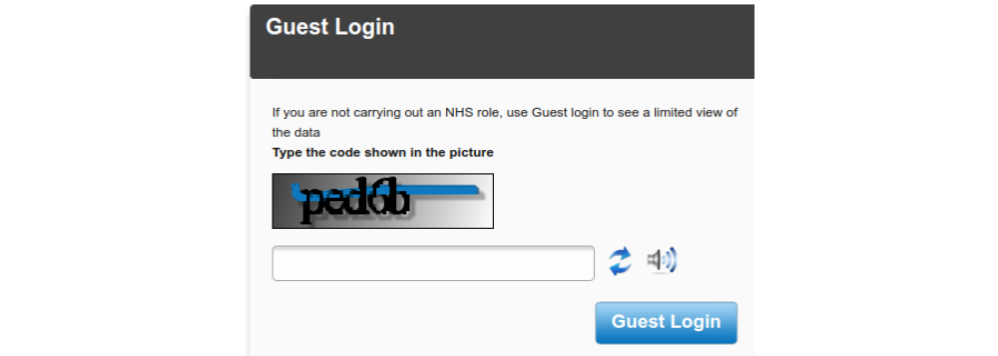
CAPTCHA form for National Health Service Business Services Authority Practice Detailed Prescribing Information dataset.

The version that we have used since 2017 is known as Practice Detailed Prescribing Information (PDPI) and is published by NHSBSA on their Information Services portal [13]. The decision to switch to using that dataset was driven by its timeliness: the monthly release is available much sooner than the practice level prescribing data. However, it is difficult to use. First, the dataset is only accessible after completing a CAPTCHA, which means automating the download process is impossible: every month a software engineer has to manually fill out a form (Figure 1).

Second, although documentation is provided for PDPI, the documentation is incomplete: it refers to fields that do not exist, and does not refer to 15 fields that do exist [14]. Finally, to our surprise, we read in a newsletter from NHSBSA that the version of the data on their Information Services portal would be replaced by a new system in December 2018 [15]. We have since established that nothing will change for the time being for end users but were surprised there was no public consultation about the possibility. Changing our systems to support a new location (and potentially format) could conceivably take several weeks and early warning for this kind of change is essential. Textbox 1 outlines some steps that could be taken to improve access to data.

**How access to data could be improved.**

- No publicly available data should be protected behind a CAPTCHA.
- Each dataset should have every field documented.
- Every resource should have a consistent location (URL or machine-readable data index) for finding current data.
- Internal reorganizations should not result in these URLs being deleted; if they are superseded, old URLs should be kept and set up to redirect to new locations.
- When there is a plan to relocate or change datasets, this should be advertised and documented well in advance.
- It should be easy to find all current prescribing data resources and to pick the most appropriate one. For example, there could be a single place listing all current prescribing data resources.

## British National Formulary Names

Each prescription is identified by a “BNF Code”: this is typically 15 characters long and uniquely identifies a presentation of a drug. For example, the code for Tramadol HCl 300 mg tablets is 040702040AAAMAM. To make prescribing data useful for analysts, all the British National Formulary (BNF) codes must be converted to human-readable BNF names. Data to support this are published by NHSBSA (behind another CAPTCHA) on the Information Services portal [16].

**Figure 2:**
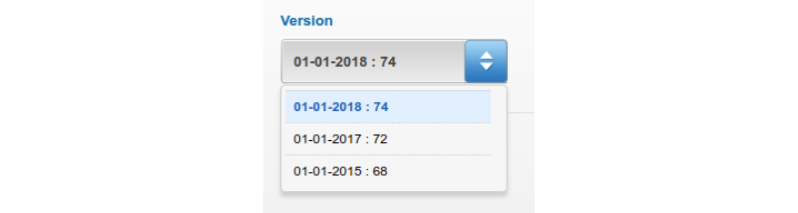
British National Formulary Code data labeled available in November 2018.

The coding scheme is based on the BNF’s old classification, which they no longer maintain themselves. Therefore, the NHS’ altered version is properly known as the “Pseudo BNF Classification” [17]. Changes to BNF coding take place throughout the year, with a large reclassification process happening every January, when some drugs are moved between BNF sections or BNF chapters, and others are given entirely new BNF codes. This reclassification process is not mentioned, let alone described, anywhere we can find on the internet. The process by which the BNF file is updated is unclear. Although we know a major revision is published every January, minor revisions are also published monthly, but a user would not know this because the data download page only refers to the January editions (Figure 2).

The fact that some BNF codes change over time makes time-based analysis of data difficult. For example, a user searching for Linaclotide, using its current BNF code, will find no prescribing before 2014. This is because the drug was moved from BNF section *1.2: Antispasmod. & Other Drgs Alt. Gut Motility* to BNF paragraph *1.6.7: Other Drugs Used In Constipation*, and its BNF code changed accordingly.

As there is no mention that such changes are possible on the internet, we first inferred this was the case following user enquiries about apparently disappearing drugs. Following direct enquiries, we now obtain a spreadsheet detailing the changes every January by emailing NHSBSA directly and apply this to the imported prescribing data. By comparing the pseudo BNF code lists each month, we have inferred that codes also sometimes change mid-year but have not yet obtained access to these individual changes on a monthly basis [18]. Textbox 2 describes some of the ways the NHS could aid public understanding of BNF code changes.

**How British National Formulary change management could be improved.**

- Published, open data should never be protected behind a CAPTCHA.
- The fact that the British National Formulary (BNF) scheme changes regularly should be documented.
- There should be a single, obvious channel for data consumers to query possible issues in the data.
- BNF code changes should be published monthly as a mapping.
- Each data release should be clearly labeled on its index page, so users know when a new version has been released; there should be a way for users to subscribe to be notified of new releases.

## Practice Data

To include patient list size in our analyses, and show practice names and addresses, we looked up extra information in a dataset provided by NHS Digital. The format of this dataset has not changed since 2015; however, we have encountered regular problems with its location changing, which has prevented us from fully automating this monthly process.

Until 2018, our procedure was to automate a search for the phrase “*Number of Patients Registered at a GP Practice*” on the NHS Digital website and then look for datasets in the list of results returned. From July onward, the data were moved to a different location. In addition, the format of the dates within the file changed between June and July. The location has changed twice more since then. All these changes mean that it is common for the code that automatically imports practice list information to break and to require manual input.

Once practice data were obtained, we encountered difficulties with data quality. In general, the data provided by NHSBSA are of a high standard. However, there is no documentation for several known recurring errors and no way to report and correct them systematically.

For example, it is important to know whether an institution is a standard GP practice or a different kind of institution (eg, a homeless service or a drop-in center). However, in the data provided, there is a small but significant number of obvious errors in coding, such as classification of care homes [19] and violent patient services [20] as standard settings. When we queried these problems, we were informed that errors can only be corrected by CCGs themselves; however, they were unable to provide us with CCG contact details to contact these organizations ourselves systematically and notify them of the need to make these corrections to their own data. It is also unclear if there is any part of the NHS that considers itself responsible for maintaining accurate data in this area.

As a final example, this problem is further compounded by list size data that regularly appears to contain fictional values. Sometimes we identify practices that have prescribing at improbable levels, far exceeding their total number of patients [21]. These may be data entry errors but sometimes appear to be caused by an unusual design of the data specification: when a new practice is registered, the NHSBSA proforma states that a list size *must* be given, which can be “nominal” but must be under 100 [22]. Our interpretation of this is that any list size of less than 100 must be considered arbitrary and cannot be relied on. This interpretation may be wrong but is our best guess in the absence of documentation. The best practice for data management is that missing values should be clearly coded as such. Textbox 3 contains further suggestions for aiding consumption of practice list size data.

**How practice data quality and accessibility could be improved.**

- All data should be published in a predictable format and location.
- “Nominal” values should not be used: missing values should be clearly coded as “missing.”
- Where there are systemic issues with data quality, these should be documented.
- There should be a clearly documented and centralized system for reporting and correcting errors in the data.
- Data stewards should take responsibility for collecting error reports and aim to correct them.

## Clinical Commissioning Group Codes, Boundaries, and Membership

To analyze data at a CCG level, we need to aggregate the per-practice data up to CCG level. The source data provide a CCG for each row, so this is straightforward for contemporary data.

## Maps

**Figure 3:**
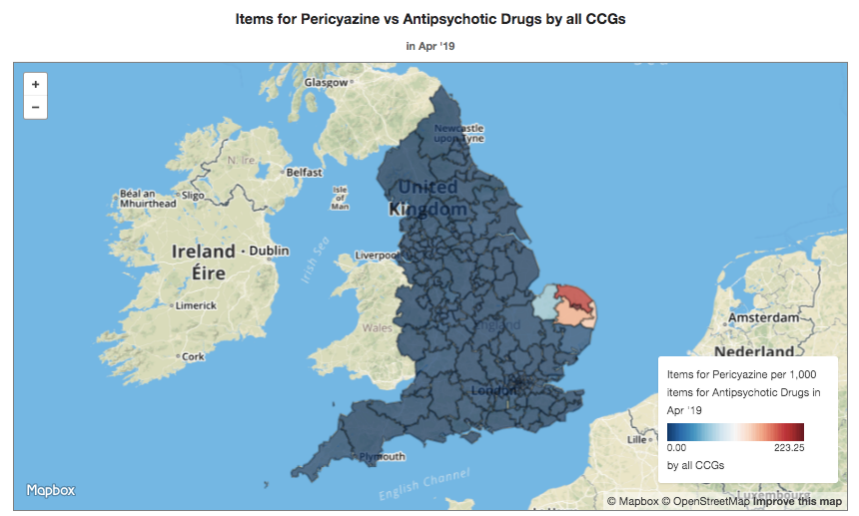
Prescribing of pericyazine as a proportion of all antipsychotics across all Clinical Commissioning Groups in England, as displayed on OpenPrescribing.net.

We show CCG boundaries on maps in various places in OpenPrescribing.net: an example in Figure 3 shows unusually high prescribing of pericyazine, a very unusual antipsychotic, in Norfolk. Until 2017, we obtained the map data from NHS England [23]. This was updated occasionally; on one occasion it was supplied in a different format from usual, so we had to alter our software accordingly. In 2017 it became apparent that this map was no longer being kept up to date with changes in CCGs. We found a new file provided by the Office for National Statistics (ONS) [24], which was up to date. For some time, both maps were available. During this period, the NHS England map was 2 years out of date, and with no indication there was a more up-to-date map in a different location. Eventually, the NHS England version disappeared from the website. The current ONS map appears to be accurate but, unlike the NHS England one, does not include a CCG identifier, so a separate data file is needed to associate CCGs with their boundaries; again, the file is supplied with no clear indication of where such a mapping can be found.

## Clinical Commissioning Group Practice Membership Changes

Our per-practice data provides a practice’s CCG membership for the current month. However, historic analysis is complicated by the fact that practices often change CCGs, CCG boundaries sometimes change, and CCGs often merge. In 2017, for example, the boundary between NHS Cumbria and NHS Morecambe Bay changed. We were able to infer from the data that 32 practices moved to Morecambe Bay as a result [25].

The problem that a practice may move between CCGs is addressed in the OpenPrescribing.net software by projecting the practice’s current CCG membership back in time: for example, a prescription dispensed in 2012 is allocated to whatever CCG that practice currently belongs to. This works well in most cases but becomes complicated when a practice has closed. In the case of Cumbria in April 2017, 5 practices had closed before the boundary change; these are, therefore, not reflected in current CCG membership data. This leaves the problem of which CCG to attribute them to: their patients have not disappeared, just moved, but it is impossible to find out or infer where they were moved to because the information about what happens to a practice’s patient list on closure is not available as data.

Our own research has established that when a practice closes (or merges), it must fill out at least two nearly identical forms to notify the prescription pricing division of NHSBSA [26] and Primary Care Support England (PCSE) [27]. We requested any data resulting from both these forms in Freedom of Information (FOI) requests to the respective organizations [28,29]. NHSBSA informed us that “Prescriber and practice/cost centre amendments are only held as paper forms,” and PCSE eventually supplied us with a spreadsheet that appeared to bear little relationship to real closures [30]. As a result, every April (when boundary changes happen), our developers have to make educated guesses about which contemporary CCGs the patients of a closed practice now belong to, and at a practice level, there is nothing we can do to amend the data correctly [25].

Textbox 4 provides some suggestions on how the quality and accessibility of mapping data could be improved.

**How National Health Service mapping data could be improved.**

- Map files should be published in a single, easily found, permanent location to a regular schedule.
- They should be published alongside (or indicate the location of) files supporting mapping to standard National Health Service (NHS) clinical commissioning group (CCG) codes as used in prescribing data.
- Their format should stay constant over time where possible.
- Practice merger and closure data should be published, showing where and when practice lists have transferred.
- Even if this is not possible, the problem of tracking historic prescribing behavior via practice codes should be clearly documented.
- We are unclear as to the value for the NHS of a system that requires CCGs to notify NHS England of practice changes but then leaves the information on paper.

## What Does “Quantity” Mean?

A single row of prescribing data includes a column denoting the quantity of the item dispensed. For example, in the case of paracetamol tablets, a “quantity” of 25 means that 25 *tablets* were dispensed. This field is used in most of our analyses. For example, our price-per-unit tool [6] identifies possible savings by comparing the price of dispensed drugs between practices nationally, at a “quantity” level. However, a consistent and precise definition of what “quantity” means for each product has been elusive [31]. For example, the NHS Digital glossary [17] defines quantity as follows:

The quantity of a drug dispensed is measured in units depending on the formulation of the product, which is given in the drug name. Where the formulation is tablet, capsule, ampoule, vial etc. the quantity will be the number of tablets, capsules, ampoules, vials etc. Where the formulation is a liquid, the quantity will be the number of millilitres. Where the formulation is a solid form (eg. Cream, gel, ointment), the quantity will be the number of grammes.

However, this definition is not sufficiently precise for use in statistical analyses. For example, it is not obvious if a foam should be classified as a liquid or a solid. Further extensive investigation uncovered the existence of a “standard quantity unit” field for every product, which defines the property precisely. However, it can be found only in one place, the monthly prescription cost analysis spreadsheet [32] and is not mentioned anywhere outside that dataset. This useful column was removed without warning in the data from December 2018 onward.

Even when the standard quantity unit for a presentation is known, the definition of quantity sometimes varies, for example, between “dose” and “pack.” During development of our price-per-unit tool, we found a number of products where the highest price was orders of magnitude outside the normal range [33]. Items dispensed in packs of 56, for example, were sometimes being recorded as a quantity of both 1 or 56.

An NHSBSA glossary has this to say on the matter: “Where a product is packed in a 'special container'...*in some circumstances* [our emphasis] these items show quantity as the number of units supplied ie 1 or 2 even though a pack may contain 56 tablets” [31].

It is not clear from this statement if variation in the meaning of “quantity” for a single presentation is intentional, or accidental. We raised specific examples with NHSBSA, and this led to some of these figures being corrected retrospectively, but in other cases, we were told “work is under way to review this and agree a way forward.”

Errors in data are inevitable and to be expected. Overall, the NHSBSA dataset is remarkably free of errors. However, as analysts we need to understand where errors are and, if they are systematic, where, and how often we can expect them to appear. The detailed investigative analysis required to understand these data delayed the launch of our price-per-unit savings tool by several months. This kind of delay has real-world effects; published peer-reviewed data show that the tool saves CCGs millions of pounds a year [6]. Textbox 5 summarizes some ways the meaning and quality of published datasets could be improved.

**How the meaning and quality of datasets could be made clear.**

- By default, all prescribing data used internally at National Health Service Business Services Authority should be made available and described in 1 place.
- All data should be accompanied by clear, user-focused documentation about the meaning of each field.
- Where there are known problems with the data, these should be documented clearly and transparently.

## How Can We Contact Practices by Email?

During 2017, we set out to conduct a simple, low-cost randomized trial: we notified GPs of cost-saving and quality improvement opportunities in their prescribing and are currently measuring the impact of this notification on their behavior. The intervention was split between 3 methods of communication: letter, email, and fax. We assumed there must be at least one central NHS database of practices’ email addresses; for example, NHS England emails a monthly GP practice bulletin to GP practices. We knew there might be problems making this public, but we were also surprised by how difficult it was to find out if the database existed at all.

First, we checked WhatDoTheyKnow, a publicly accessible archive of requests made under the FOI Act for any past FOI requests for GP practice contact information. We found NHS England had refused a similar request for practice information stating that “NHS England does not hold information in relation to your request” [34]. We knew this was unlikely to be correct, so we sent a new request asking specifically for the contact details for the GP practice bulletin, which we knew was emailed to practices by NHS England [35]. The response acknowledged the existence of a list and recognized there is “a general public interest in the release of such information in-line with NHS England’s commitment to openness and transparency.”

However, the request was refused under 2 of the allowed exemptions in the FOI Act. The first was section 40 (an exemption relating to personal information). They argued it would be unfair to staff, who had signed up for one purpose, to be contacted for another purpose. The second was section 43 (an exemption relating to commercial interests). This is apparently because some of the GP email addresses had been purchased by NHS England from a third party under a license that forbids the NHS to share the information.

Having failed with one database we knew to exist, we made requests to every public body that might hold a database of GP email addresses. We preemptively included an argument that section 40 should not apply as these are work email addresses. All were refused, with similar arguments to those from NHS England or invoking section 21 (the information was already available—which is incorrect) or stating that they did not hold the information. The responses are summarized in Table 1.

**Table 1:** Summary of responses to Freedom of Information requests for general practitioner’s email addresses.

Body	Reasons for not supplying the data
Department of Health and Social Care	Information not held [36]
NHS^a^ England (new request)	S21 [34]
NHS England (follow-up)	S40 [34]
Medicines and Healthcare Products Regulatory Agency	S21, S40, S43 (their own commercial interests) [37]
NHS Business Services Authority	S21, S40, S43 [38]
NHS Digital	S21 and information not held [39]

^a^NHS: National Health Service.

In our view many of the responses gave the impression of an organization actively seeking ways to refuse releasing this information. NHSBSA argued that providing email addresses would damage commercial interests because it decreases security: “The e-mail addresses could be used by cyber criminals to target practices, CCGs etc. If such an attack was successful it could result in financial loss and/or loss of patient data.” This strikes us as an extremely unrealistic concern. The notion that hiding information intrinsically increases security has been long debunked in the security research community, where it is known disparagingly as “security through obscurity;” and in any case, the email addresses are all available through commercial data providers. Furthermore, most GP practices would expect to be contactable through email by their patients.

We were eventually able to run the randomized controlled trial (RCT) but only at greatly increased cost. We sent FOI requests to all 201 CCGs [40], of which 29 agreed to share at least some emails. We also wrote code to download data from the NHS Choices website. Finally, we combined our results with the commercial dataset that we purchased. In the end, we got emails for 27.0% (190/703) of practices from the NHS Choices website, 7.9% (56/703) from our FOI requests, and the remainder from a commercial provider. Notably, the email addresses purchased from a commercial vendor were apparently higher quality than those available directly from NHS resources: where we obtained an email address commercially, 18% (86/474) of practices accessed an email link versus 11% (23/211) for email addresses we obtained from other sources.

In summary, the reasons given for not supplying email addresses were inconsistent and sometimes hard to fathom. We believe the section 40 exemption (that it is unfair to individuals to release this data) is overused: given these are work addresses for managers, there would be a strong case for their release, based on current FOI guidelines. At the very least, given that 33% (66/201) of CCGs were willing to provide email addresses, the exemption is very unevenly interpreted and applied. We also understand that the vast majority of practices have a generic nhs.net inbox, which would certainly be exempt from section 40, but a list of even these email addresses is apparently unavailable.

Ultimately, it should not be difficult for researchers, health professionals, or indeed the public to have a way to contact any GP practice by email; and until this is the case, it should not be difficult (as it currently is) for a data consumer to establish definitively that there is no such resource. The problems we had assembling these data delayed the start of our RCT by several months. This delay indirectly affects care, as there is currently limited research available about how information is best disseminated through the NHS. We also note that the Secretary of State for Health and Social Care has prominently promoted the principle of using emails first, rather than letters, to communicate in the NHS. This is made harder if NHS organizations themselves are failing to make email addresses easily available, or actively blocking access. Textbox 6 summarizes the steps we suggest could be taken to address this problem.

**How to make it easier to contact general practitioners.**

- There should be a contact database for general practitioner practice managers, including email addresses, which is available to the public. Currently the choice to make an email address public is taken by practices alone.
- In the meantime, to save time and effort on the part of users of the data and NHS bodies, the fact that it is currently unavailable should be clearly documented in a single place, with an explanation, and suggestions for alternative sources.

## What Are Historic Drug Tariff Prices?

**Figure 4:**
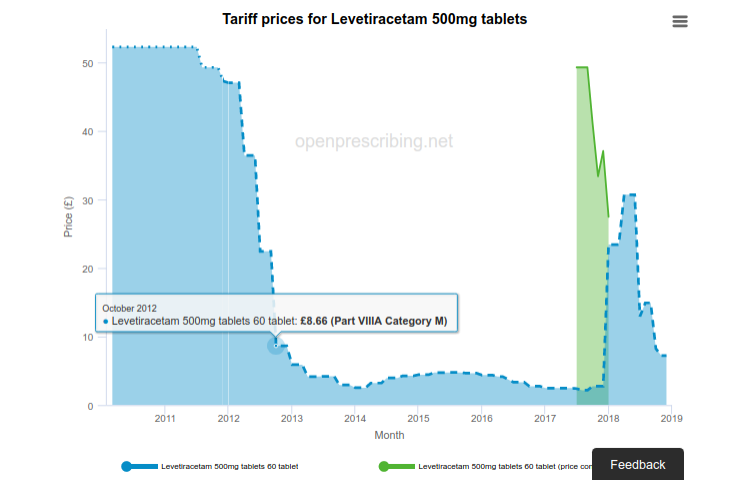
Tariff prices and projected cost impact of price changes for Levetiracetam as on OpenPrescribing.net.

For prescriptions written in primary care in England, the NHS reimburses community pharmacies for the medicines they purchase. The reimbursement price for generic medicines is set monthly by the Department of Health and Social Care (DHSC) in the NHS *Drug Tariff*. Price changes in the Drug Tariff are a major source of variation in costs for CCGs. In addition, the DHSC grants temporary excess rates in the form of price concessions every month, which can have sudden and unexpected effects on costs. We set out to create a tool on OpenPrescribing.net for tracking these changes over time and modelling the cost impact of new price concessions for each CCG using their past prescribing behavior (Figure 4). By processing the data as soon as it is published, we can provide email alerts to CCG budget holders warning them of upcoming price pressures.

The tool was relatively easy to build. However, we could only build it after a large amount of difficult research and manual data editing. First, the data must be combined from spreadsheets found on 2 totally different websites, although both ultimately originate from DHSC. Second, each spreadsheet refers to the information in a different way, and they both provide separate files of data each month whose formats change over time. Finally, they are archived inconsistently, which makes it hard to locate historic data.

Finding the most recent data is relatively easy. Drug Tariff data are provided by the NHSBSA in a single location, which provides monthly spreadsheets for the last 2 years of the Drug Tariff [41]. In these data, every product is supplied with a unique Systematized Nomenclature of Medicine (SNOMED) code, which we can use to map to our prescribing data. Price concession data are published by the Pharmaceutical Services Negotiating Committee [42]; however, these data are supplied without SNOMED codes, which means the products must be matched to prescribing data by name only. This is hard to automate because the names often deviate in subtle ways (eg, “sq cm” vs “square cm”).

To find earlier datasets for previous years, we turned to the NHSBSA FOI archive [43]. The Web page has no search function, so we had to review all the content in the archive manually. We found 2 ZIP files of Excel spreadsheets in 2 different FOI requests [44,45]. These had gaps in their coverage. With educated guesses around filename and location, we were able to fill in these gaps by finding “hidden” files on the internet: files that were available on the public internet and on the BSA website but which were not indexed in any search engines and were not linked to from anywhere. Having assembled the data, we had 49 files, in several different formats, which we standardized and combined using formulas, heuristics, and Excel plugins [46]. Once this was done, we were able to build a tool and automate monthly updates; however, because of the lack of SNOMED codes in the price concessions data, for most months we still had to manually match 1 or 2 products to the prescribing data. Textbox 7 summarizes some of the ways by which access to this (and similar linked datasets) could be improved.

**How price concessions data could be made easier to use.**

- When archived data that are part of an already published time series are made available, the data should be published alongside the time series, not left in Freedom of Information requests.
- Any data export process of relatively small datasets should ideally involve producing a single file of all the data each month. This avoids the problem of formats changing through time.
- All data should be provided with identifiers.
- All shared data should be indexed or indexable.
- It is surprising that the price concession data are not already combined with Drug Tariff data somewhere in the National Health Service.
- Price concessions should be mentioned in documentation wherever Drug Tariffs are mentioned.

## Discussion

### Summary

As illustrated, although several NHS datasets are “open” by the narrowest definition, the NHS commonly breaches the principles of the Open Government White Paper and barely meets other best practice criteria such as the Berners-Lee 5-Star scale for open data. Collectively, the barriers described in this paper represent a substantial block to the development of new data-driven tools aiming to improve the quality, safety, and efficiency of NHS care. These barriers can be broadly divided into 4 areas: problems accessing the data, problems understanding the data, problems processing the data, and problems communicating with the NHS about the data. These imply 4 solutions: better curation, better documentation, better change management, and better dialogue with users. Here we summarize the barriers and offer some concrete suggestions of how the situation could be improved.

### Better Curation

As documented in previous sections, there is a very substantial problem with curation of information in the NHS. Datasets are collected and shared at considerable expense but are then commonly undiscoverable or poorly indexed; they move location unpredictably, and often an interested user cannot establish whether a given dataset exists at all. The NHS England Data Catalogue [47] is largely unstructured: this means users must already know what they are looking for before they can find it. It also contains numerous older datasets, with no way for the user to deduce whether the dataset itself has been abandoned, with no further updates, or if only the catalog record is out of date. Catalogs are commonly divided by organization: this assumes that all users understand the complex organizational structure of the NHS and are able to predict whether a dataset is owned by NHS England, NHS Digital, NHS Improvement, NHSBSA, or some other organization. The problems we describe are often caused by ineffective automation. We propose 2 approaches for the NHS: “proactive curation” and “reactive curation.”

Proactively, the NHS should invest in manually curating the data it already shares. This would entail detailed strategic input from experts in information management and librarianship; here we offer some brief principles. First, this curation should be done by people, with individuals or teams owning a particular topic area. Second, these teams must include domain experts already working within the relevant NHS organizations who understand the data. Third, instead of separate silos of data arranged by NHS organization, there should be a single location with links to all NHS data; and there should be confidence that all the data relevant to the topic are indexed in that one place as per best practice and government guidance [7]. Finally, these resources should be tagged in multiple dimensions, including their clinical domain, topic, and technical characteristics. In our view, the ideal model would be “topic-based guides” that are clearly branded, owned, and maintained by a single team; focused on adding new resources as they become available; and ensuring that data resources do not disappear or move.

Reactive curation also offers substantial benefits but at much lower cost. In short, where users are actively working on datasets, and they report to the NHS that something is missing, out of date, or poorly documented, then these errors, omissions, and shortcomings should be addressed and corrected. In short, there should be a simple means for users to report errors in the existing catalogs and for these errors to be corrected.

We can see no reason why any NHS resource should be behind a CAPTCHA, but if this is unavoidable, the reasons for this choice should be robustly documented and forewarning given in the catalog.

### Better Documentation

In the previous sections we have documented numerous cases where NHS datasets are hard to interpret because of poor documentation and where the NHS has not been reactive to questions around poor or absent documentation. Documentation is challenging and time-consuming. However, good documentation brings clear thinking: an organization that cannot produce or share documentation on the data it holds is unlikely to be working effectively with that data internally.

We think there is room for the “proactive and reactive” model described above. Proactively, every dataset should be accompanied by documentation that explains its context (how and why it is used in the NHS), its provenance, the meaning of each field, how often it is updated, and any known issues with the data. At minimum, datasets that are regularly downloaded, used, or enquired about should be prioritized for this best practice. Reactively, the NHS should respond to queries, and there should be easy routes for users to give feedback on ambiguities or errors in the documentation. However, the NHS should also work more strategically with end users of the data, as this is where the true value of that dataset is often realized: documentation should ideally be developed in the open, in collaboration with data consumers, to ensure it is current and relevant.

### Better Management of Change Over Time

As documented in previous sections, there are substantial problems with NHS datasets changing structure and format over time, often without those changes being documented. Often these changes are trivial: the names of the columns in a 2-way table or their order. However, every time the format of a dataset changes then there is a material consequence, for every end user: the pipelines for importing and processing data will break, the fault must be discovered, and developers must work around it. Commonly, there seems to be no technical reason for the changes we have seen in NHS datasets: it is likely that these changes simply reflect carelessness, or a lack of interest and knowledge about how the data are being used and processed by end users.

In an ideal world, data formats would never change; however, occasional changes are inevitable. Therefore, clear communication of changes is vital. We suggest that every dataset should be accompanied by a change log in its catalog entry. This change log should describe the nature of each format change. Crucially, there should also be clear documentation of the reasons why the change has happened, as this is likely to act as an informal feedback system, prompting NHS staff to think through whether the change is really necessary. There should be a way for consumers of the data to subscribe to updates and receive advance notice of these changes and to provide feedback where changes have happened without documentation, prompting the change log to be updated.

A related issue is stability of data structures over time when working with older datasets. Users often want to automatically retrieve and process not only current data but historic data. In doing so, they hit 2 problems: finding all historic files and resolving the format differences between them. To aid discovery, the naming conventions for data (eg, “title, date”) should be documented in the same way as the data structure itself, and remain stable over time, to support automated retrieval. Where the formats of shared datasets must change, but the NHS holds historic data internally in a consistent format, then for all but the largest datasets, we suggest a bulk export of all historic data in the most current format should ideally be provided. Again, following the principle of transparency, where bulk exports are not possible, this should be mentioned and explained in the documentation.

### Better Dialogue Between Data Producers and Data Consumers

We have returned repeatedly to the importance of better communication between data producers and consumers, if only as a means to reactively prioritize work around curation and documentation. In our view, this 2-way communication is vital: producers should be able to notify consumers of important changes in the data, and consumers should be able to notify producers of bugs in the data or ask questions. It is important to note here that good dialogue cannot be driven by a positive attitude alone: we have had many very positive interactions with single individuals in various NHS organizations who have been very helpful, but individuals can change jobs, or go on holiday, and finding the right person to talk to often relies on personal networks or sheer determination.

A strategic approach to dialogue requires good systems and formal structures. In short, the NHS needs a single place for users to ask questions about data, with the answers recorded and searchable in the public domain. This should be well publicized, open, public, and linked to liberally from across the NHS online estate. Given the NHS’ general commitment to transparency [3,4], it would make sense for data producers in the NHS to borrow from best practices developed in the open source software movement, where source code is available to everyone, anyone can suggest edits, and anyone can report bugs. Each question answered in public will then be added to the commons of knowledge that can easily be accessed via any internet search engine. This platform should be curated by an NHS employee who has the authority to pursue questions on users’ behalf and expect answers within a reasonable time frame. Over time, a database of questions and answers could evolve into a series of topic-based guides, written in collaboration with the user community. Many of the problems faced by those working with NHS data will already have been solved several times over by analysts within the NHS or third parties elsewhere.

None of this requires custom software and could all be provided through standard, widely used, free, open services such as GitHub and GitLab. End users could contribute bug reports to the documentation; they could ask questions through the bug tracking systems; everyone could see everyone else’s input, helping raise standards and awareness; and the data producers could reply on built-in notification features to push feeds of updates to the end users. The most important part of solving this problem is not software but staff expertise and time. By reducing the friction between the 2 sides of the data exchange, better uses of data will emerge.

For clarity, this is not a “blue skies” or challenging suggestion. This is a standard way of working outside of the NHS, and it is how our own team works: we document every step of our problem solving publicly, in our closed and open “issues” on GitHub, which now number over 1000 [48]. Anyone working with NHS data who has been blocked by the same technical barriers we have hit can find our solutions—and the reasoning leading up to them—simply by using a search engine.

### Conclusions

Releasing data under open licenses was the starting point for open data and the open government movement, in which the United Kingdom has been a global leader. However, in our experience, the implementation of these open principles in the NHS has been absent or flawed, with poor documentation, poor curation, and poor dialogue presenting substantial barriers to innovation. We have chosen to spend time documenting these issues at length; many third parties confronted with similar barriers will either give up, concluding a service as impractical, or quietly expend substantial resource and effort on workarounds. This in turn will increase the cost of delivery, block innovation, and divert resources that should be spent on producing better services for clinicians, commissioners, and patients.

There is currently substantial appetite for better use of data and software in the NHS [49]. This will only happen if the system engages constructively and technically with the individuals and teams who actually use that NHS data on a daily basis. We hope this paper will stimulate further dialogue between data providers and end users; we welcome feedback and further examples of both good and poor practice, and we are keen to engage, on both the details and broader strategic issues, with all members of the NHS and wider community.

## Abbreviations

BNF: British National Formulary
CCG: clinical commissioning group
DHSC: Department of Health and Social Care
FOI: Freedom of Information
GP: general practitioner
NIHR: National Institute for Health Research
NHS: National Health Service
NHSBSA: National Health Service Business Services Authority
ONS: Office for National Statistics
PCSE: Primary Care Support England
PDPI: Practice Detailed Prescribing Information
RCT: randomized controlled trial
SNOMED: Systematized Nomenclature of Medicine
SQL: Structured Query Language
